# KIAA1199 as a potential diagnostic biomarker of rheumatoid arthritis related to angiogenesis

**DOI:** 10.1186/s13075-015-0637-y

**Published:** 2015-05-29

**Authors:** Xinyu Yang, Pengcheng Qiu, Bingbing Chen, Yaoyao Lin, Zhonghao Zhou, Renshan Ge, Hai Zou, Jianmin Wang, Jianguang Wang

**Affiliations:** Department of Biochemistry, School of Basic Medical Sciences, Wenzhou Medical University, Wenzhou, China; Department of Medicinal Chemistry, School of Pharmaceutical Sciences, Wenzhou Medical University, Wenzhou, China; Department of Rheumatology, Jiamusi Central Hospital, Jiamusi, China

## Abstract

**Introduction:**

Our previous proteomic study on fibroblast-like synoviocytes (FLSs) derived from the synovial tissues found that the expression of KIAA1199 was higher in rheumatoid arthritis (RA) patients than in healthy controls. The aim of this study was to examine the biological function of KIAA1199 and evaluate its clinical diagnosis value in RA.

**Methods:**

The over-expression of KIAA1199 was verified by quantitative real-time polymerase chain reaction (qPCR), Immunohistochemistry, Immunofluorescence and enzyme linked immunosorbent assay (ELISA) in inactive and active RA patients and healthy controls. The effect of KIAA1199 expression on FLSs proliferation, angiogenesis and related pathway were analyzed by MTT, cell migration, tube formation, chorioallantoic membrane (CAM) assay, qPCR and western-blotting after KIAA1199 knockdown and over-expression.

**Results:**

The verification results show the up-regulation of KIAA1199 in RA patients at mRNA and protein level as compared to that in healthy controls. ELISA and receiver operator characteristic (ROC) analysis shows that KIAA1199 concentration in serum, synovial fluid and synovial tissues could be used as dependable biomarkers for the diagnosis of active RA, provided an area under roc curve (AUC) of 0.83, 0.92 and 0.92. Sensitivity and specificity, which were determined by cut-off points, reached 72% 84% and 80% in sensitivity and 80%, 93.3%, 93.3% in specificity, respectively. Moreover, KIAA1199 also enhance the proliferation and angiogenesis of synovial membrane, and KIAA1199/ PLXNB3/ SEMA5A/CTGF axis may be a newly found pathway enhancing cell proliferation and angiogenesis.

**Conclusion:**

KIAA1199 may be a potential diagnostic biomarker of RA related to angiogenesis.

**Electronic supplementary material:**

The online version of this article (doi:10.1186/s13075-015-0637-y) contains supplementary material, which is available to authorized users.

## Introduction

Rheumatoid arthritis (RA) is a chronic inflammatory disease characterized by progressive joint damage. The pathogenesis of RA is complex and thought to be mediated by various mechanisms. Early events in RA disease progression are defined by hyperplasia of the synovial membrane, influx of leukocytes and inflammatory cells. Activated fibroblast-like synoviocytes (FLSs) in the lining layer of the synovial membrane are among the dominant cell types involved in pannus formation, and pannus is a key player in joint destruction [1,2]. Angiogenesis is now recognized as a key event in the formation and maintenance of the pannus in RA [3,4].

*KIAA1199* gene is a member of the large transmembrane protein of the KIAA family with more than 1000 amino acids [5] discovered about 10 years ago. Human *KIAA1199* gene is located on chromosome 15q25.1 segment, which encodes a 150 kDa protein originally described as an inner ear protein [6]. KIAA1199 was found to have a G8 domain [7] and two GG domains [8]. Although the basic function of KIAA1199 remains unknown, an inverse correlation between the expression level of KIAA1199 and disease stage/5-year survival rate suggests that KIAA1199 may be associated with cancer progression [9]. It was also demonstrated that KIAA1199 was over-expressed in excessively proliferated cancer tissues, including those from gastric cancer [9], breast cancer [10-12] and colon cancer [13-18]. In addition, our previous proteomic study on FLSs derived from the synovial membrane also found that KIAA1199 expression in RA patients was significantly higher than in healthy controls [19], but the biological function and mechanism of action of KIAA1199 in RA remain unknown.

The aim of the present study was to verify the over-expression of KIAA1199 mRNA and protein in the serum, synovial fluid and synovial tissues obtained from patients with active and inactive RA and healthy controls, explore the effect of KIAA1199 on FLSs proliferation and angiogenesis by MTT, cell migration, tube formation and chorioallantoic membrane (CAM) assay after KIAA1199 knockdown and over-expression.

## Methods

### Patients and primary culture of FLS cells

The serum was obtained from 44 RA patients, 15 osteoarthritis (OA) patients, 15 ankylosing spondylitis (AS) patients and 15 normal subjects. Knee synovial fluids and synovial tissues were from 44 RA patients undergoing synovectomy or joint replacement surgery and 15 normal subjects undergoing high-level amputations in Shanghai Changhai Hospital and Shanghai Guanghua Hospital (Shanghai, China). RA patients were further categorized as a group with active RA (n = 25) and a group with inactive RA (n = 19) depending on the elevation of disease activity score in 28 joints (DAS28) (inactive RA: DAS28 < 3.2; active RA: DAS28 > 3.2); DAS28 score correlates closely with clinical parameters of RA disease activity [20]. Patients fulfilled the 1987 American College of Rheumatology criteria for the diagnosis of RA [21]. The clinical data of the patients are shown in Table 1. Serum and synovial fluid were stored at −80°C immediately after centrifugation at 12,000 rpm. One part of synovial tissues was stored at −80°C, another part was isolated enzymatically according to the method previously described [19]. All FLSs of passages three to five were used for the experiment. This study was approved by Shanghai Changhai Hospital ethics committee (CHEC2013-194), with informed consent from all the participants concerned.

**Table 1:** **Demographic characteristics of patients and normal subjects**

	**Normal**	**Inactive rheumatoid arthritis (disease activity score in 28 joints <3.2)**	**Active rheumatoid arthritis (disease activity score in 28 joints >3.2)**	**Osteoarthritis**	**AS**
**Number**	15	19	25	15	15
**Age, years**	47.2 ± 9.91	53.64 ± 6.32	57.56 ± 9.75	54.65 ± 8.43	38.75 ± 9.34
**Male/female, n**	6/9	8/11	10/15	7/8	8/7
**Serum C-reactive protein, mg/dl**	0.19 ± 0.09	0.20 ± 0.07	1.37 ± 0.89	0.18 ± 0.04	0.38 ± 0.17
**Disease activity in 28 joints, score**	0.94 ± 0.41	2.57 ± 1.35	5.38 ± 3.93	NA	NA
**Erythrocyte sedimentation rate, mm/h**	9.83 ± 3.06	28.35 ± 13.41	47.36 ± 29.41	10.57 ± 2.44	36.9 ± 13.52
**Duration of disease, years**	NA	4.1 ± 2.7	8.3 ± 4.3	6.4 ± 2.9	10.6 ± 5.8
**Bath ankylosing spondylitis disease activity index, score**	NA	NA	NA	NA	7. 85 ± 5.24
**Non-steroidal anti-inflammatory drug usage, %**	NA	78.3	81.3	NA	46.6
**Disease-modifying anti-rheumatic drug usage, %**	NA	63.5	65.1	NA	38.9

Values are expressed as mean ± SD unless stated otherwise. NA, not applicable.

### RNA preparation and quantitative real-time PCR analysis

Total RNA was extracted from the synovial tissues or FLS cells by Trizol reagent (Invitrogen, Carlsbad, CA, USA), precipitated with isopropanol and dissolved in DEPC-treated distilled water. The concentration of total RNA was determined by Eppendorf BioPhotometer Plus (Hamburg, Germany). Total RNA (2 μg) was then treated with RNase-free DNase (Invitrogen, Carlsbad, CA, USA) before the first-strand cDNA was generated using the random hexamer primer provided in the first-strand cDNA synthesis kit (MBI Fermantas, Vilnius, Lithuania). Specific amplification was performed using the primers of *KIAA1199* genes (forward primer: 5′ TGC TGC CCG GGT ATT CAA AT 3' and reverse primer: 5′CGT CCA CTC CAC GTC TTG AA 3′), *plexnB3* genes (forward primer: 5′ ACC CAG GTC AAG GAG AAG GT 3′ and reverse primer: 5′ GTC TTC GTC CGA TAG GGT CA 3′), *sema5A* genes (forward primer: 5′ GCT CCT TCC ACA AGA AGT GC 3′ and reverse primer: 5′ CAA GCT GCT TCC AAG AAT CC 3′), *ctgf* genes (forward primer: 5′ TGG AGT TCA AGT GCC CTG AC 3′ and reverse primer: 5′ GTA ATG GCA GGC ACA GGT CT 3′) and *β-actin* (forward primer: 5′ATGG TGG GTA TGG GTC AGA AG 3′ and reverse primer: 5′TGG CTG GGG TGT TGA AGG TC 3′) used as an internal control for determining the cell number and metabolic status. Quantitative real-time PCR (ABI7300, Applied Bio-systems, Carlsbad, CA, USA) was done with SsoFast EvaGreen supermix PCR kit (Bio-Rad, Hercules, CA, USA). A total of 40 cycles of PCR was performed for 15 s at 95°C, and 60 s at 60°C. The relative expression of each target gene compared with *β-actin* was calculated using the 2^-ΔΔCt^. All reactions were conducted in triplicate.

### Immunohistochemical analysis

Synovial tissues were fixed overnight in 4% paraformaldehyde, embedded in paraffin, and sectioned in 5- to 8-μm intervals. In brief, sections on slides were de-paraffinized, re-hydrated, antigens unmasked by incubating in target retrieval solution at 95°C for 30 minutes, permeabilized in 0.1% Triton-X100 for 5 minutes, blocked with 10% chicken serum in TBST for 45 minutes, and incubated with KIAA1199 monoclonal antibody (sc-164775, Santa Cruz, CA, USA) at 1/20 at 37°C overnight. Subsequently, a peroxidase 3,3-diaminobenzidine (DAB) detection system (SK6333-2; Sangon Biotech, Shanghai, China) was applied according to the manufacturer’s instructions. The sections were observed under a fluorescence microscope.

### Immunofluorescence microscopy

FLS cells grown on glass coverslips were washed with PBS, fixed at room temperature with 4% paraformaldehyde (20 minutes ), permeabilized with 0.5% Triton-X 100 (10 minutes), and blocked with 10% normal goat serum (30 minutes). They were then incubated with KIAA1199 monoclonal antibody (sc-164775, Santa Cruz, CA, USA) and CTGF (ab6992, abcam) overnight at 4°C, and then with secondary antibodies (rabbit anti-goat IgG Dylight™ 549 conjugated and donkey anti-rabbit IgG Alexa Fluor 488 conjugated , catalog number: BM8505 and BMJ8106, Bio Mart, New Delhi, India) for 45 minutes at 37°C. The cells were covered with 4',6-diamidino-2-phenylindole (DAPI)-Vectashield mounting medium (236276, Roche, Switzerland), and images were captured on an epifluorescence microscope (Leica, Wetzlar, Hessen, Germany) equipped with Leica Application Suite V3.3.0 software.

### Enzyme-linked immunosorbent assay for human KIAA1199

KIAA1199-specific antigens were detected in serum, synovial fluid and synovial tissues extraction samples from patients with active RA, inactive RA, OA and AS, and healthy subjects. The homogenate was centrifuged in a micro-centrifuge for 5 minutes at 3,000 g, and 100 μl diluted supernatant (1:50 with incubation buffer) was incubated at room temperature in a microtiter plate coated with KIAA1199 monoclonal antibody (sc-164775, Santa Cruz, CA, USA). After incubation, washing and addition of a detection antibody coupled to horseradish peroxidase, the substrate was added and incubated, followed by addition of a stop solution. The absorption rate was determined at an optical density of 450 nm. All reactions were conducted in triplicate.

### KIAA1199 knockdown and over-expression

KIAA1199 knock-down experiments were performed by transfecting KIAA1199-specific siRNA (100nM). The siRNA sequence targeting KIAA1199 was 5′ -AAA CAU UGA AAU AUU CGC CAU GCU C- 3′ and 5′ -UUG ACA AGG AGG CCA AGA CAG UGG U- 3′. Scrambled siRNA was 5′-UUU UCG CUG CGC CAA CCU CTT-3′ and 5′-AUA AGG GAA CGU GAG CGC GTT −3′. KIAA1199 over-expression experiments were performed by transfecting pcDNA3.2DEST-cloned KIAA1199 open reading frame (100 nM), which was supplied freely by Dr Marra (Institute of Molecular Cancer Research, University of Zurich, Zurich, Switzerland) [15].

### Western blotting analysis

An equal amount of protein extract (50 μg per lane) was separated by SDS-PAGE of 10% polyacrylamide, and electrotransferred onto polyvinylidene fluoride (PVDF) membranes. The membranes were incubated for 1 h with a blocking solution containing 0.1% Triton X-100, 5% nonfat milk in PBS. The KIAA1199 monoclonal antibody (Abnova, H00057214-M01), PLXNB3 (Santa Cruz, sc-46240), SEMA5A (sc-67953, Santa Cruz, CA, USA), CTGF (R&D Systems, AF660, Minneapolis, MN, USA) were then added. The membranes were incubated overnight at 4°C, and then with the appropriate horseradish peroxidase-conjugated secondary antibody at room temperature for 1 h. The filter was then incubated with the substrate and exposed to radiographic film. All reactions were conducted in triplicate.

### Cell proliferation assay

Cell proliferative activities were examined using FLSs. Cells were seeded onto 96-well plates (1 × 10^4^ cells/well) for 24 h and treated with a fresh culture medium containing various concentrations of KIAA1199 siRNA duplex (50, 100, 200 nM) or scrambled siRNA for 72 h at 37°C. The proliferative capacity of FLSs was determined by the MTT-based cell proliferation and viability assay system according to the manufacturer’s instructions. The results showed that the viability of KIAA1199 siRNA-treated FLSs decreased significantly in a dose-dependent manner (data not shown), and 100-nM KIAA1199 siRNA duplex was used in this study. FLS cells were seeded onto 96-well plates for 24 h and transfected with scrambled siRNA, KIAA1199 siRNA duplex (100nM), pcDNA3.2DEST and pcDNA3.2DEST-KIAA1199 (100 nM) for 24 h, 48 h, 72 h and 96 h at 37°C by Lipofectamine 2000 (Invitrogen, Carlsbad, CA, USA) according to the manufacturer’s protocol. The proliferative capacity of FLSs was determined by the MTT-based cell proliferation and viability assay system. The differences in absorbance were compared in vector control and KIAA1199-transfected cells. The assay was performed in triplicate.

### Cell migration and endothelial tube formation assays

Transwell motility assays were performed by 6.5 mm Transwell® with 8.0-μm pore polycarbonate membrane filters (Corning Corp, Corning, NY, USA). Human umbilical vein endothelial cells (HUVEC) transfected with scrambled siRNA, KIAA1199 siRNA duplex (100 nM), pcDNA3.2DEST and pcDNA3.2DEST-KIAA1199 (100 nM) were cultured to 85% to 95% confluence, subjected to serum starvation for 24 h before cell migration assay, dissociated by incubation with trypsin-EDTA, washed twice with PBS, and counted using a hemocytometer. Then 600 μl medium was added to each lower chamber of the 24-well transwell for 24 h at 37°C. Inserts (the upper chambers) were then placed in the wells. Cells (1 × 10^5^ in 300 μl serum-free media) were added to each upper chamber and incubated for 6 h. Non-migrating cells were removed with a cotton swab. Cells that migrated to the lower phase of the upper chamber were then fixed in methanol for 30 minutes and stained with crystal violet for 3 minutes at room temperature. Excess stain was removed with distilled water, and the chambers were air-dried. Pictures were taken under the microscope and the cell number was quantified by software Image-Pro. The assay was performed in triplicate.

The tube formation assay was performed as follows: 24-well plates were pre-coated with Matrigel and incubated at 37°C to promote gelling. HUVEC were added to each well transfected with scrambled siRNA, KIAA1199 siRNA duplex (100 nM), pcDNA3.2DEST and pcDNA3.2DEST-KIAA1199 (100 nM). After 6 h incubation, the plates were fixed with 4% paraformaldehyde and a blinded observer assessed the morphology of the tubes. Tube-like structures were quantified by counting the number of intersections between branches of the endothelial cell networks in the whole field. The assay was performed in triplicate.

### Chorioallantoic membrane assay

Angiogenic activity of KIAA1199 was assayed on CAM as described by Takigawa *et al.* [22]*.* Embryonic CAM were treated on day 7 with scrambled siRNA, KIAA1199 siRNA duplex (100 nM), pcDNA3.2DEST and pcDNA3.2DEST-KIAA1199 (100 nM) absorbed on sterile Whatman GB/B glass fiber filter disks (6 mm in diameter; Reeve-Angel, Clifton, NJ, USA). The disks prepared with 20 μl of factor were placed upside down on windows that had been made in the eggshells on day 7 of incubation. The embryos were examined 3 days later under a stereomicroscope. ImageJ 2.43 s was used to calculate the vascular area and CAM area.

### Statistical analysis

Data analysis involved estimation of the mean and SD using SPSS 17.0. The Shapiro-Wilk method and histograms were used to test whether the data were normally distributed. The Levene method was used to test homogeneity of variance. Two sets of data that met the normal distribution and homogeneity of variance were analyzed by independent samples *t*-test. The Kruskal-Wallis and Mann-Whitney non-parametric tests were used to compare interassay differences in data that did not meet the normal distribution or the homogeneity of variance. Inspection level *P*-values <0.05 were considered statistically significant.

## Results

### Elevated expression of KIAA1199 in RA patients

**Figure 1:**
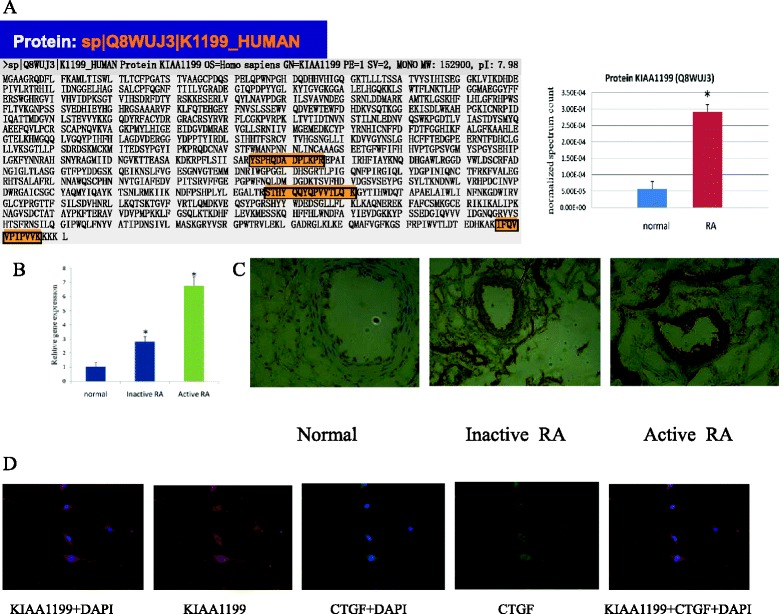
Identification and verification of KIAA1199 expression in rheumatoid arthritis (RA) patients and normal subjects. **(A)** Identification the over-expression of KIAA1199 in RA fibroblast-like synoviocyte (FLS) cells than in normal FLS cells from synovial membrane by automated 2D-Nano-LC-ESI-MS/MS. The left panel shows amino acid coverage of the identified KIAA1199 sequence; yellow, appraisal sequence of amino acids. The right panel is the normalized spectrum count in RA and normal subjects; *P <0.001. **(B)** Verification of the expression of KIAA1199 was performed in synovial tissues from 19 patients with inactive RA, 25 with active RA and 15 normal subjects by qPCR. Results were normalized for the amount of β-actin as internal control. Experiments were performed at least in triplicates; bars represents mean ± SD; *P <0.05 compared with normal. **(C)** Immunohistochemical staining of KIAA1199 was performed on synovial tissues from patients with inactive or active RA and normal subjects. The KIAA1199 protein was over-expressed in vascular endothelium in active and inactive RA synovial membrane compared to normal synovial membrane. **(D)** Immunofluorescence microscopy of KIAA1199 and CTGF was performed in FLS cells from RA patients. Result shows KIAA1199 and CTGF were co-expressed in FLS cells, were either not expressed or only very weakly expressed in the nucleus, and were strongly expressed in the cytoplasm and membrane of FLS cells.

Our previous comparative proteomic study results showed that KIAA1199 protein was 5.19 times over-expressed in RA FLSs [19]. The results of automated 2D-Nano-LC-ESI-MS/MS identification are shown in Figure 1A and Additional file 1. In this study quantitative real-time PCR was performed to confirm whether there was a real difference between RA and normal synovial tissues in the expression of KIAA1199. Compared to healthy subjects, the KIAA1199 mRNA expression was 2.77-fold and 6.62-fold up-regulated in inactive RA and active RA patients than in healthy subjects, respectively (Figure 1B), which was consistent with the change in the previous proteomic study. The immunohistochemical analysis shows the expression of KIAA1199 was significantly higher in the vascular endothelium of synovial membrane in active RA and inactive RA than in normal synovial membrane (Figure 1C). Immunofluorescence microscopy shows that KIAA1199 and CTGF proteins were co-expressed in FLS cells, and they were either not expressed or only weakly expressed in the nucleus, and were strongly expressed in the cytoplasm and membrane of FLS cells (Figure 1D).

### Correlation between KIAA1199 and disease activity

**Figure 2:**
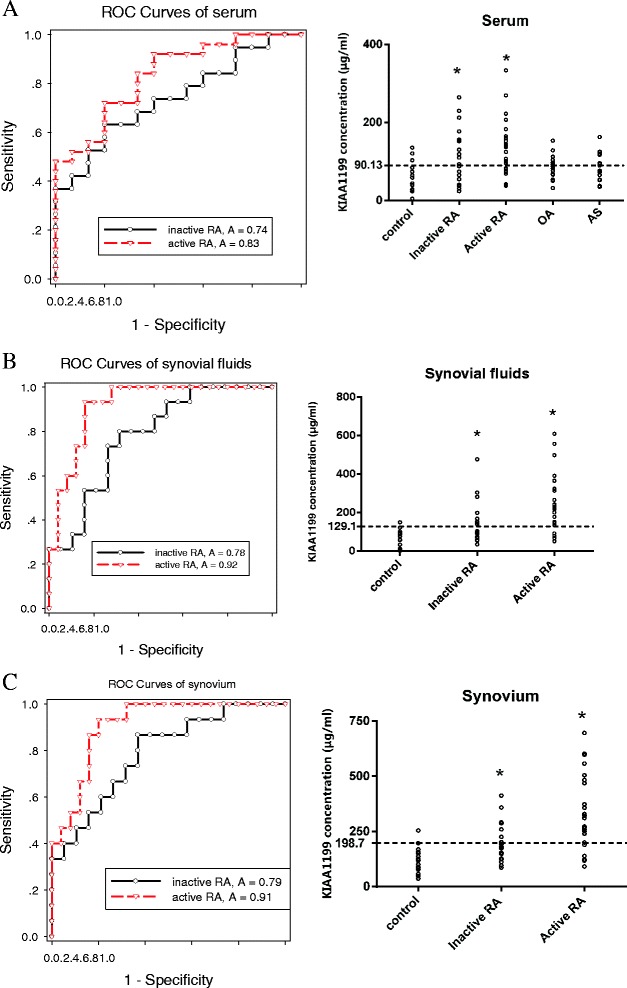
Diagnostic value of evaluation of KIAA1199 in rheumatoid arthritis (RA) determined by receiver operator characteristic (ROC) curve analysis. **(** **A** **)** ROC curve analysis of KIAA1199 in serum for diagnosis of RA. The area under the curve (AUC) for inactive RA-healthy controls (HC) and active RA-HC was 0.74 and 0.83, respectively. A cutoff value of 90.13 μg/ml diagnosed active RA at the sensitivity of 72% and the specificity of 80%, respectively. **(** **B** **)** ROC curve analysis of KIAA1199 in synovial fluids for the diagnosis of RA. The AUC for inactive RA-HC and active RA-HC was 0.78 and 0.92, respectively. A cutoff value of 129.1 μg/ml diagnosed active RA at the sensitivity of 84% and the specificity of 93.3%, respectively. **(** **C** **)** ROC curve analysis of KIAA1199 in the synovium for the diagnosis of RA. The AUC for inactive RA-HC and active RA-HC was 0.79 and 0.91, respectively. A cutoff value of 198.7 μg/ml diagnosed active RA at the sensitivity of 80% and the specificity of 93.3%, respectively. All experiments were performed at least in triplicates; *P <0.05 compared with control. OA, osteoarthritis; AS, ankylosing spondylitis.

Knowing that DAS28 score is an indicator for measuring clinical disease activity, RA patients were divided into an active or inactive group according to DAS28 score (inactive RA: DAS28 < 3.2; active RA: DAS28 > 3.2). The abundance levels of KIAA1199 in the serum, synovial fluid and synovial tissues of patients with inactive and active RA and healthy subjects were determined by ELISA. KIAA1199 protein was expressed in human serum, synovial fluid and synovial tissue, while the expression of KIAA1199 protein was increased in the RA group compared to the healthy group (*P* <0.05). There were no significant differences in KIAA1199 in serum among patients with OA or AS, or healthy controls, and the concentration of KIAA1199 in RA was different from that of OA and AS. Therefore the concentration of KIAA1199 can be used as a biomarker of RA. The levels of KIAA1199 in serum, synovial fluid and synovial tissue were significantly elevated in patients with active RA as compared to those with inactive RA. Interestingly, the number of samples that were KIAA1199-positive was significantly greater in the active RA than that in the inactive RA group (18 versus 11 in the serum, 21 versus 9 in the synovial fluid, and 20 versus 9 in the synovial tissues).These data suggest that the level of KIAA1199 in serum, synovial fluid and synovial tissues was correlated with the disease activity and was associated with the process of RA (Figure 2).

### ROC curve analysis determined the diagnostic value of KIAA1199 for RA

In order to determine the diagnostic value of KIAA1199 for RA, we conducted a receiver operator characteristic (ROC) curve analysis of KIAA1199. The result reveals that the areas under the ROC curve for the serum, synovial fluid and synovial tissues from inactive RA were 0.74, 0.78, and 0.79 (range 0.7 to 0.8), respectively, indicating medium diagnostic value. The area under the ROC curve for serum, synovial fluid and synovial tissues from active RA were 0.83, 0.92 and 0.91, respectively, indicating high diagnostic value. Cutoff values of 90.13, 129.1 and 198.7 μg/ml were able to detect individuals with active RA at the sensitivity of 72%, 84% and 80%, respectively, and specificity of 80%, 93.3% and 93.3%, respectively (Figure 2). The results show better diagnostic value of KIAA1199 in synovial fluids and synovial tissue in RA.

### The effect of KIAA1199 on FLS cells proliferation

**Figure 3:**
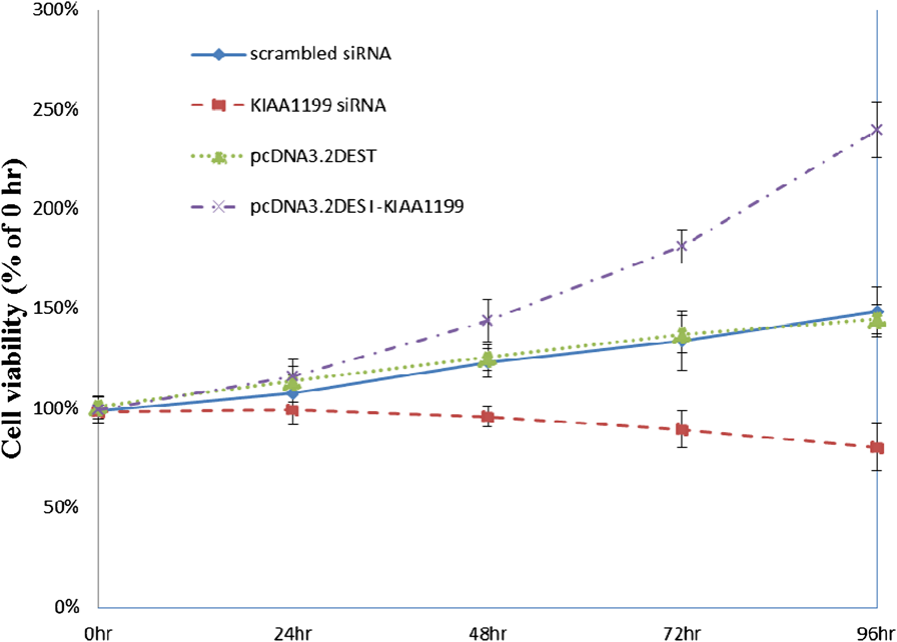
The effect of KIAA1199 expression on fibroblast-like synoviocyte (FLS) cell proliferation detected by MTT assay. FLS cells isolated from synovial tissue were transfected with scrambled siRNA, KIAA1199 siRNA duplex, pcDNA3.2DEST and pcDNA3.2DEST-KIAA1199 for 24 h, 48 h, 72 h and 96 h at 37 °C. Cell viability was determined by MTT. All experiments were performed at least in triplicates.

The proliferation of synovial membrane is now recognized as a key event in RA progression. To determine the effects of KIAA1199 expression on FLS cell proliferation, the MTT assay was performed on FLS cells treated with scrambled siRNA, KIAA1199 siRNA duplex, pcDNA3.2DEST and pcDNA3.2DEST-KIAA1199 for 24 h, 48 h, 72 h and 96 h at 37°C. The MTT results showed that the viability of FLSs treated with KIAA1199 siRNA decreased significantly after KIAA1199 knock-down and increased after KIAA1199 over-expression; significant differences were observed between every time point examined, indicating a linear change in proliferation with treatment time, *P* <0.05. These data support the assumption that KIAA1199 may be an inducer of FLSs proliferation (Figure 3).

### The effect of KIAA1199 on angiogenesis

**Figure 4:**
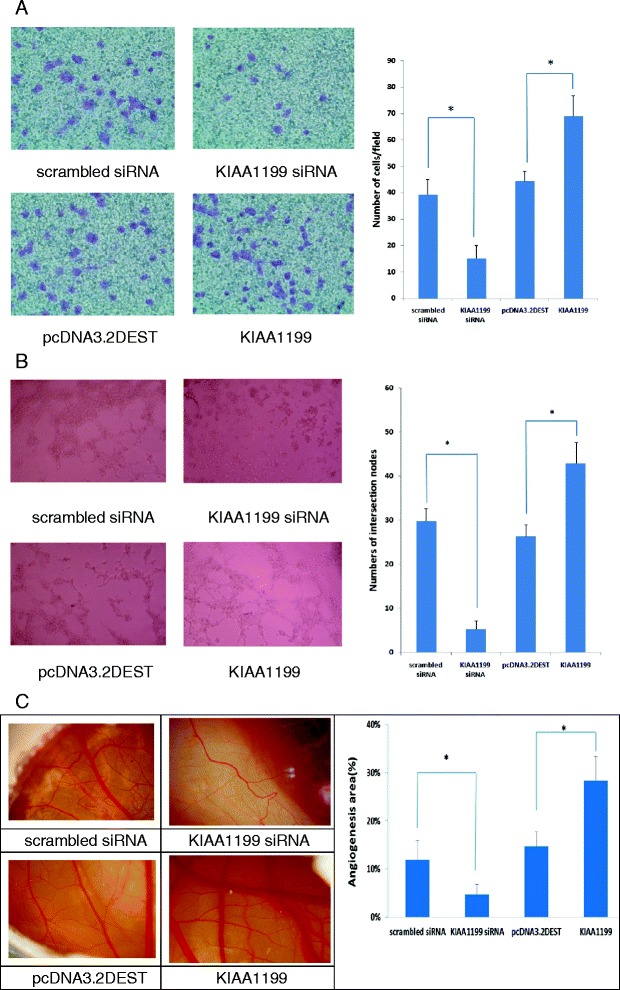
Effect of KIAA1199 expression on angiogenesis. **(** **A** **)** Cell migration assay. Human umbilical vein endothelial cells (HUVEC) transfected with scrambled siRNA, KIAA1199 siRNA duplex, pcDNA3.2DEST and pcDNA3.2DEST-KIAA1199 were plated onto the top of the transwell, allowed to migrate for 4 h, then rinsed, fixed, stained, and counted. Left panel: HUVEC on the undersurface of a filter, magnification × 40; right panel: number of HUVEC per field. **(B)** Tube formation assay: 24-well plates were pre-coated with Matrigel (diluted 1:2 in H2O) and incubated at 37°C to promote gelling. HUVEC were seeded on Matrigel-coated plates, treated with scrambled siRNA, KIAA1199 siRNA duplex, pcDNA3.2DEST and pcDNA3.2DEST-KIAA1199 for 6-8 h, tube formation observed using a phase-contrast inverted microscope, and photographs were taken from each well. The number of intersections between branches of assembled endothelial cell networks was counted in the whole field. **(** **C)**. Chorioallantoic membrane (CAM) assay, embryonic CAM was treated on day 7 with scrambled siRNA, KIAA1199 siRNA duplex, pcDNA3.2DEST and pcDNA3.2DEST-KIAA1199. After incubation for 3 days CAM was examined under a stereomicroscope, and photographs taken (left panel). ImageJ 2.43 s was used to calculate the vascular and CAM areas. Bar graph: ratio of vascular area to CAM area (right panel). All experiments were performed at least in triplicates, values are presented as mean ± SD; *P <0.05 compared with control (*significant differences).

Angiogenesis is now recognized as a key event in the formation and maintenance of the pannus in RA. To further evaluate the effects of KIAA1199 expression on cell angiogenesis, the transwell and tube formation assays were performed on HUVEC cells treated with scrambled siRNA, KIAA1199 siRNA duplex, pcDNA3.2DEST and pcDNA3.2DEST-KIAA1199 for 6 h at 37°C. The transwell assay result shows KIAA1199 knock-down reduced the migration ability and KIAA1199 over-expression increased the migration ability of HUVEC cells, *P* <0.05 (Figure 4A). The tube formation experiment shows a similar result to the transwell assay, *P* <0.05 (Figure 4B). The above *in vitro* findings allowed us to investigate the angiogenic activity of KIAA1199 *in vivo.* First, we carried out a CAM assay. After 3 days treatment of scrambled siRNA, KIAA1199 siRNA duplex, pcDNA3.2DEST and pcDNA3.2DEST-KIAA1199 on CAM of chick embryos, the formation of small vessels decreased in KIAA1199 knock-down and increased in the KIAA1199 over-expression experiment, *P* <0.05, suggesting that KIAA1199 is a requirement for angiogenesis (Figure 4C).

### Effect of the KIAA1199/PLXNB3/ SEMA5A/CTGF axis on angiogenesis

**Figure 5:**
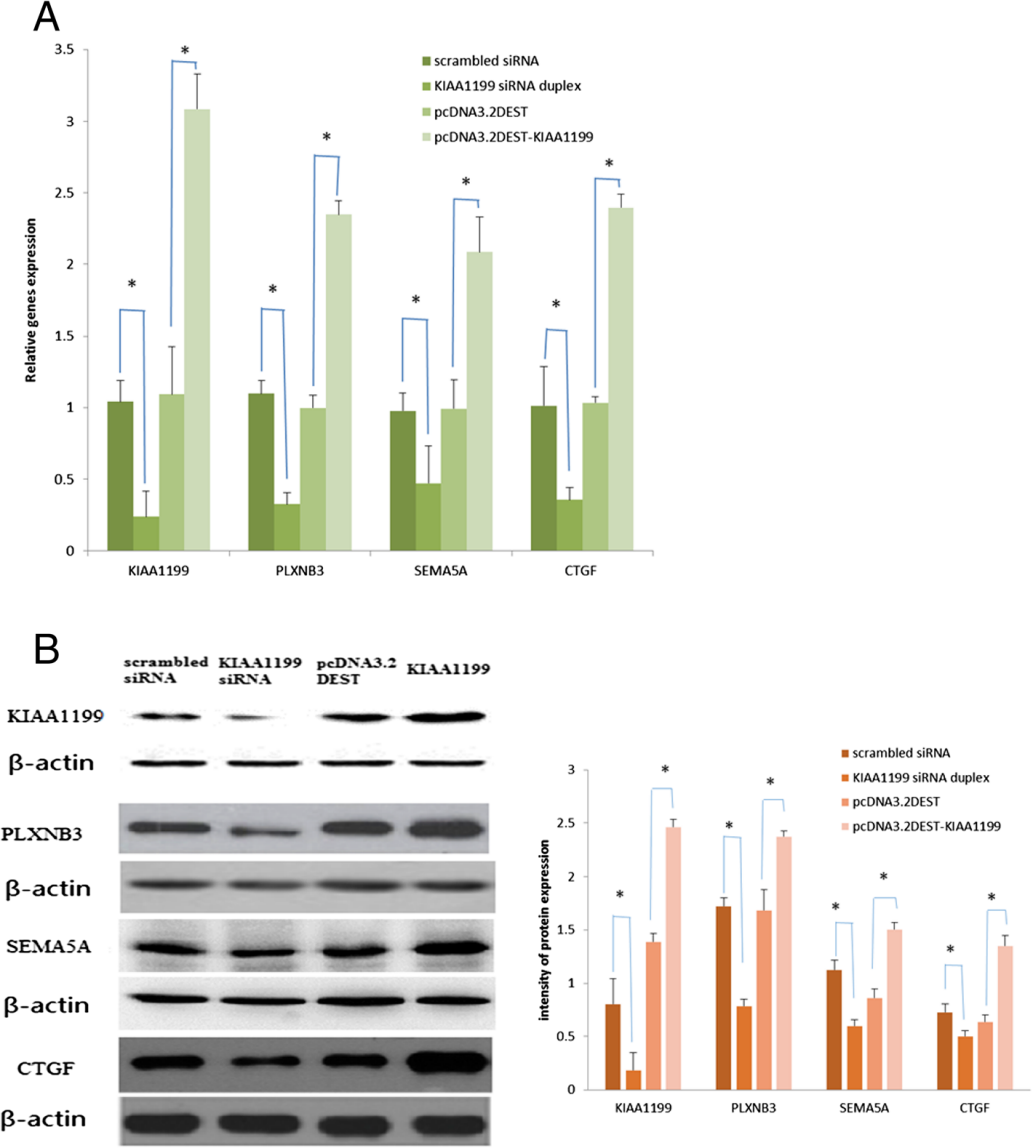
Effect of KIAA1199 knock-down and over-expression on the expression of PLXNB3, SEMA5A and CTGF in fibroblast-like synoviocyte (FLS) cells. **(** **A)**. Real-time PCR of KIAA1199, PLXNB3, SEMA5A and CTGF transfected with scrambled siRNA, KIAA1199 siRNA duplex, pcDNA3.2DEST and pcDNA3.2DEST-KIAA1199 for 24 h in FLS cells; β-actin was used as a loading control. **(** **B)**. Western blotting of KIAA1199, PLXNB3, SEMA5A and CTGF transfected with scrambled siRNA, KIAA1199 siRNA duplex, pcDNA3.2DEST and pcDNA3.2DEST-KIAA1199 for 48 h in FLS cells; β-actin was used as a loading control. All experiments were performed at least in triplicates, the data are presented as mean ± SD; *P <0.05, compared with control (*significant differences).

To clarify the pathway of KIAA1199 regulation of angiogenesis in FLS cells in RA, hypothetical-pathway-related proteins were determined by qPCR and western blotting in triplicate. We observed an decrease of 77.0%, 70.3%, 51.7%, and 64.9% in RNA levels and a decrease of 76.5%, 54.6%, 47.0%, and 30.7% in protein levels of KIAA1199, PLXNB3, SEMA5A and CTGF after KIAA1199 knock-down, and an increase of 182.6%, 135.5%, 110.3%, and 132.2% in RNA levels and an increase of 77.5%, 40.7%, 75.4%, and 112.0% in protein levels of KIAA1199, PLXNB3, SEMA5A and CTGF after KIAA1199 over-expression, respectively. This is to say, the expression difference of PLXNB3, SEMA5A and CTGF were proportional to the expression of KIAA1199 in mRNA and protein levels (Figure 5A and B).

## Discussion

A hallmark of RA is the pseudo-tumoral expansion of FLSs, which induces the pannus formation and erodes the cartilage and bone. Many observations [23] have prompted speculation that joint FLSs in RA evolve genetically to form a locally invasive and metaplastic tissue. So cytokine-enhanced FLS proliferation in RA would be a potential biomarker and has therefore been proposed as a therapeutic target by knocking down its expression.

Our previous comparative proteomic study showed that KIAA1199 was 5.19 times over-expressed in RA FLS cells as identified by automated 2D-Nano-LC-ESI-MS/MS [19]. This finding is supported by Yoshida’s result showing that the level of KIAA1199 expression in non-inflamed synovial tissues was lower than in that of rheumatoid synovial tissues as shown by real time PCR and immunoblotting (n = 3) [24]. However, this has not been confirmed in a large set of samples, neither with regard to RNA or protein levels, nor with regard to gene function. In this study, we evaluated KIAA1199 expression by quantitative RT-PCR and ELISA in 44 patients with RA (19 with inactive and 25 with active RA) and 15 healthy controls. The data were analyzed to determine the clinical significance of KIAA1199 levels in RA. It was found that the expression of KIAA1199 mRNA and protein were higher in the synovial tissues of RA patients than healthy subjects; ELISA also showed that the levels of KIAA1199 protein expression were higher in the serum, synovial fluid and synovial tissue from RA patients than healthy subjects, suggesting that the over-expression of KIAA1199 plays an important role in the pathogenesis of RA. These data not only indicate that the level of KIAA1199 expression is higher in RA but suggest positive correlation between KIAA1199 expression and the DAS28 score, an RA index correlated closely with clinical parameters of RA disease activity. ROC curve analysis indicated high diagnostic value of KIAA1199 in active RA. These data also imply that KIAA1199 may be a potential diagnostic biomarker of RA, but its role in RA procession remains elusive.

Yoshida *et al.* carried out further research on the relationship between KIAA1199 and hyaluronan metabolism, a very important polysaccharide in synovial fluid for minimizing friction between the bones. They found that the cleavage of N-terminal 30 amino acids occurs in functionally matured KIAA1199, resulting in altered intracellular trafficking of the molecule and loss of cellular hyaluronic acid (HA) depolymerization. This suggests that the N-terminal portion of KIAA1199 functions as a cleavable signal sequence required for proper KIAA1199 translocation and KIAA1199-mediated HA depolymerization. Notably, the secreted mature form of KIAA1199 showed no HA degrading activity, together supporting the idea that KIAA1199-mediated HA depolymerization occurred through rapid vesicle endocytosis. These results show that KIAA1199 protein promoted the degradation of HA, which is normal in physiological tissues and fast in inflammatory and neoplastic diseases. In osteoarthritis or RA synovial fibroblasts, the enhancement of HA metabolism is associated with increased expression of KIAA1199 [19,24-26]. These data indicate that KIAA1199 plays a key role in HA catabolism as a unique hyaluronic cadherin in the dermal and OA synovial tissues.

The over-expression of KIAA1199 has been identified in many proliferative tissues, including synovial tissues in RA. Several reports [27] support the importance of the Wnt pathway activation in FLS proliferation. Wnt signaling is now well-recognized as a critical pathway in the regulation of growth and development. Birkenkamp-Demtroder *et al*. [17] report that KIAA1199 expression is markedly reduced by inactivation of the β-catenin/T-cell factor transcription complex, the pivotal mediator of Wnt signaling. Thus, they identified KIAA1199 as a novel target of the Wnt signaling, the regulation of KIAA1199 by Wnt signaling was observed as a protein-protein interaction.

Immunohistochemical analysis in this study showed the expression of KIAA1199 was significantly higher in the vascular endothelium. Jami also revealed the involvement of KIAA1199 in breast cancer growth, motility and invasiveness by functional proteomic analysis [28]. Together these data suggest that KIAA1199 not only correlated with the proliferation of FLSs but may also be related to angiogenesis. The enhancing effects of KIAA1199 on angiogenesis were verified successfully in this study by transwell, tube formation and CAM assays after KIAA1199 knock-down and over-expression. CTGF is another over-expressed protein in RA patients identified in our previous proteomic study, which promotes the proliferation and migration of HUVEC [19]. Furthermore, the expression of CTGF was decreased or increased as KIAA1199 was knocked down or over-expressed. The question remains as to which pathway regulates the expression of CTGF by KIAA1199. Immunofluorescence microscopy of KIAA1199 and CTGF was performed in FLS cells from RA patients. The result shows that KIAA1199 and CTGF were co-expressed in FLS cells, and they were either not expressed or only very weakly expressed in the nucleus, and were strongly expressed in the cytoplasm and membrane of FLS cells. This experimental study gave us much inspiration. Nakayama found out that PLXNB3 is the only protein that can interact with KIAA1199, as identified by a yeast two-hybrid system [29]. PLXNB3 belongs to the plexin family [30,31] and is the receptor of SEMA5A, which is a membrane protein belonging to semaphorin (arm board protein) gene family with seven repeated platelet-response protein-1 domains [32]. Furthermore, there is a platelet-response protein-1 binding domain in CTGF [33], reminding us of the interaction between SEMA5A and CTGF by bioinformatics analysis. The binding of PLXNB3 and SEMA5A is involved in combination axon guidance [34], cell invasive growth [35], angiogenesis [36] and the process of cell migration [37]. Interestingly the synovial membrane also showed the characteristics of invasive growth and angiogenesis in the progression of RA. In our experiment the expression of PLXNB3, SEMA5A and CTGF are in accordance with KIAA1199 knock-down and over-expression. So we boldly assume that the KIAA1199/PLXNB3/SEMA5A/CTGF axis may accelerate the proliferation of FLS cells and activate the downstream angiogenic signaling pathways, leading to the formation of the pannus, and erode the cartilage and bone in the progression of RA.

## Conclusion

One of the interesting findings in this study is the more abundant expression of KIAA1199 in RA, and the KIAA1199/PLXNB3/SEMA5A/CTGF axis may be a newly found pathway enhancing cell proliferation and angiogenesis. In addition, KIAA1199 seems to have clinical correlation with the DAS28 and disease activity. In summary, KIAA1199 may be a potential biomarker and therapeutic target in RA.

## Additional file

Additional file 1: Figure S1. MS and MS/MS spectra of the peptide of KIAA1199. (A) MS and MS/MS spectrum of peptide STHYQQYQPVVTLQK. (B) MS and MS/MS spectrum of peptide YSPHQDADPLKPR. (C) MS and MS/MS spectrum of peptide IFQVVPIPVVK.

## Abbreviations

AS: ankylosing spondylitis
AUC: area under the receiver operator characteristic curve
CAM: chorioallantoic membrane
DAS28: disease activity score in 28 joints
ELISA: enzyme-linked immunosorbent assay
FLSs: fibroblast-like synoviocytes
HA: hyaluronic acid
HUVEC: human umbilical vein endothelial cells
OA: osteoarthritis
PBS: phosphate-buffered saline
qPCR: quantitative real-time polymerase chain reaction
RA: rheumatoid arthritis
ROC: receiver operator characteristic
siRNA: small interfering RNA
