# Supplementary material for: KIAA1199 as a potential diagnostic biomarker of rheumatoid arthritis related to angiogenesis
Source: Arthritis Res Ther. 2015 May 29;17(1):140. doi: 10.1186/s13075-015-0637-y (PMC4448531; doi:10.1186/s13075-015-0637-y)
Supplement: Additional file 1: Figure S1. — MS and MS/MS spectra of the peptide of KIAA1199. (A) MS and MS/MS spectrum of peptide STHYQQYQPVVTLQK. (B) MS and MS/MS spectrum of peptide YSPHQDADPLKPR. (C) MS and MS/MS spectrum of peptide IFQVVPIPVVK. [file 13075_2015_637_MOESM1_ESM.doc]

A.

MS

MS/MS

B.

MS

MS/MS

C.

MS

MS/MS

Figure S1 MS and MS/MS spectra of the peptide of KIAA1199. A. MS and MS/MS spectrum of peptide STHYQQYQPVVTLQK. B. MS and MS/MS spectrum of peptide YSPHQDADPLKPR. C. MS and MS/MS spectrum of peptide IFQVVPIPVVK.
